# Diarrheas in Infancy

**Published:** 1904-09

**Authors:** 


### Diarrheas in Infancy.

Rotch (*Amer. Med.*) regards the treatment of these conditions as still largely empirical, but believes it can usually be determined whether the large or small intestine is affected. The best success is obtained if the treatment is given by mouth, when the small intestine is the seat of disease, and by rectum, when the large intestine is affected. He also differentiates the group of cases with marked fermentation and little local lesion and ileocolitis class of cases when the lesions are more or less decidedly marked. No specific remedy or intestinal antiseptic has been found to absolutely kill the organisms or neutralise their toxins. Clear the intestines of bacteria by laxatives, support the strength, combat nervous symptoms, and hyperpyrexia are the chief indications. Of the drugs he recommends bismuth.

The following combination has been recommended after the alimentary canal is free of all irritants:

| | | | |
|---|---|---|---|
| ℞ | Bismuthi subnitratis | ʒii. | 8 |
| | Salol | gr. xvi. | 1 |
| | Aquæ cinnamomi | ʒi. | 30 |
| | Aquæ, q. s. ad | ℥ii. | 60 |

M. Sig.—Shake well. Teaspoonful in water every two hours.

For diarrhea in infants, with green stools:

| | | | |
|---|---|---|---|
| ℞ | Hydrargyri chloridi mitis | | |
| | Pulveris ipecacuanhea, aa | gr. i. | .06 |
| | Sacchari lactis | gr. xx. | 1.30 |

M. Chart No. x. Sig.—One powder three times a day.

—*Med. Recorder*

